# d-Serine produces antidepressant-like effects in mice through suppression of BDNF signaling pathway and regulation of synaptic adaptations in the nucleus accumbens

**DOI:** 10.1186/s10020-021-00389-x

**Published:** 2021-10-15

**Authors:** Zhenzhen Chen, Zhenyu Tang, Ke Zou, Zhihong Huang, Liuer Liu, Yuanjian Yang, Wei Wang

**Affiliations:** 1 Department of Neurology, The Second Affiliated Hospital of Nanchang University, Nanchang, 330006 Jiangxi China; 2 Jiangxi Mental Hospital of Nangchang University, 43 Shangfang Road, Nanchang, 330029 Jiangxi China

**Keywords:** d-Serine, Depression, Nucleus accumbens (NAc), Brain-derived neurotrophic factor (BDNF), Long-term depression (LTD)

## Abstract

**Objective:**

d-Serine is a crucial endogenous co-agonist of N-methyl-d-aspartate receptors (NMDARs) in the central nervous system and can affect the function of the brain derived neurotrophic factor (BDNF) system, which plays an essential role in modulating synaptic plasticity. The current study aimed to systematically evaluate the role and mechanisms of d-serine in depressive behavior in nucleus accumbens (NAc).

**Methods:**

d-Serine concentration in the chronic social defeat stress (CSDS) model in NAc was measured using high-performance liquid chromatography (HPLC). The antidepressant-like effects of d-serine were identified using forced swim test (FST) and tail suspension test (TST) in control mice and then assessed in CSDS model. We applied social interaction and sucrose preference tests to identify the susceptibility of CSDS model. Western blotting was further performed to assess the changes of BDNF signaling cascade in NAc after CSDS and d-serine treatment. The BDNF signaling inhibitor (K252a) was also used to clarify the antidepressant-like mechanism of d-serine. Moreover, d-serine effects on synaptic plasticity in NAc were investigated using electrophysiological methods.

**Results:**

d-Serine concentration was decreased in depression susceptible mice in NAc. d-Serine injections into NAc exhibited antidepressant-like effects in FST and TST without affecting the locomotor activity of mice. d-Serine was also effective in CSDS model of depression. Moreover, d-serine down-regulated the BDNF signaling pathway in NAc during CSDS procedure. Furthermore, BDNF signaling inhibitor (K252a) enhanced the antidepressant effects of d-serine. We also found that d-serine was essential for NMDARs-dependent long-term depression (LTD).

**Conclusion:**

d-Serine exerts antidepressant-like effects in mice mediated through restraining the BDNF signaling pathway and regulating synaptic plasticity in NAc.

## Introduction

Depression is a mental disorder characterized by notable, persistent, and life-threatening mood disorders, and it is expected to be a leading cause of disability worldwide by 2030, according to published reports from the World Health Organization (He et al. 2019). It has far-reaching social and economic ramification (Jin et al. 2019). In the past few decades, there has not yet been a highly efficient drug for treating depression (Li et al. 2020). Therefore, developing more reliable antidepressants with less adverse effects is highly demanding.

BDNF is distributed throughout the central nervous system (Arora et al. 2020). Currently, a leading hypothesis for depression suggests that BDNF signaling pathway is intimately involved in depression pathophysiology (Caroleo et al. 2019). Phosphorylation and activation of cAMP response element-binding protein (CREB) are induced by BDNF binding to tyrosine kinase B (TrkB) receptor on the cell membrane (Bai et al. 2019). CREB is a crucial transcription factor in the brain that regulates the biosynthesis of numerous pro-survival proteins, including BDNF (Alhusaini 2020). We have demonstrated that the level of activity of BDNF signaling pathway is increased in NAc of CSDS mice, whereas chronic antidepressant treatment could reverse these pathological changes (Jiang et al. 2014). These researches manifest that suppression of NAc BDNF signaling pathway could offer a novel therapeutic approach to depression.

BDNF plays an essential role in modulating synaptic plasticity (Tomassoni-Ardori et al. 2019; Kowiański et al. 2018). Depression development has been ascribed to dysfunction of the reward pathway, in which NAc plays a key role (Lorsch et al. 2018). Chronic stress has been recorded to cause drastic neurochemical changes in NAc, leading to depressive phenotypes (Shirayama and Chaki 2006). Our research has confirmed that synaptic adaptability in NAc is the key to mediate depression (Li et al. 2018; Jiang et al. 2013). NMDAR-LTD in NAc as prime regulators in the remodeling of excitatory synapses. d-Serine is a crucial endogenous co-agonist of NMDARs in the central nervous system and has been recorded to influence BDNF system's function. d-Serine elevation can improve depression-related behaviors (Otte 2013; Wei et al. 2017; Malkesman et al. 2012). CSDS exposure was demonstrated to destabilize d-serine in NAc (Wook Koo et al. 2016). This destabilization constitutes continued molecular adaptability of excitatory synapses to chronic stress, leading to the corresponding development of behavioral plasticity. Accumulating evidence reveals the impairment of NMDAR-dependent LTD in NAc of animal models of depression (Belujon and Grace 2014).

In short, BDNF system and synaptic plasticity in NAc are closely related to depression, and d-serine affects BDNF system's function and synaptic plasticity in NAc. However, there is no detailed study on the role of BDNF system and synaptic plasticity in NAc in d-serine-mediated antidepressant mechanism. Accordingly, we herein aim to systematically evaluate d-serine's role on depression behaviors mediated by BDNF system and in LTD in NAc.

## Materials and methods

### Animals

Adult male C57BL/6J mice (8 weeks old) and male CD1 mice (12 weeks old) were obtained from the Experimental Animal Center of Medical College, Nanchang University. Before being used, mice were housed under standard conditions (12 h light/dark cycle; lights on from 7:00 to 19:00; 23 ± 1 °C ambient temperature; 55 ± 10% relative humidity; bedding replacement twice a week) 1 week with free access to food and water. All animal care and experimental protocols were conducted according to the National Institutes of Health Guidelines for the Care and Use of Laboratory Animals and the guidelines of the Animal Care and Use Committees of Nanchang University, China.

### Drugs

d-Serine, K252a, and fluoxetine were purchased from Sigma (Alhusaini 2020). d-Serine and fluoxetine were dissolved in 0.9% saline. K252a was dissolved in DMSO to 25 mM and ethanol to 5 mM. The dosages of d-serine (2 µg/perside, 5 µg/perside), fluoxetine (5 µg/perside), and K252a (5 µg/perside) were chosen based on previous reports with some modifications(Jiang et al. 2012). d-Serine, K252a, and fluoxetine were stereotaxically injected into bilateral NAc. The repeated drug treatment of mice was performed once daily between 9:30, and 10:30 am.

### Treatment schedules

Repeated drug therapy was administered to CSDS mice once daily between 9:30 am and 10:30 am for 14 days. The doses chosen were based on the behavioral consequences and previous records (Xu et al. 2017). All these compounds were stereotactically injected into the bilateral nucleus accumbens (NAc). The corresponding vehicle was provided to control mice in the parallel volume.

### Intranucleus accumbens (NAc) infusions

Briefly, C57BL/6J mice were anaesthetized with isoflurane (3% isoflurane for induction and 2% isoflurane for maintenance) and fixed in a stereotaxic apparatus (Kopf, Tujunga, CA), with the anaesthetizing effects evaluated by muscle relaxation, slow corneal reflex, and no skin pinch reaction. The scalp was cut, and the skull was exposed using 75% alcohol and 1% H_2_O_2_. The cannulas were implanted into the bilateral NAc according to bregma: anteroposterior, + 1.5 mm; mediolateral, + 1.0 mm; dorsoventral, − 4.5 mm (Jiang et al. 2013). The cannula was cemented in place, and the incision was sutured. The animals were allowed to recover for at least 5 days after surgery. Osmotic minipumps were designed to deliver bilateral microinjection of different amounts of d-serine (2 µg/perside; 5 µg/perside), K252a (5 µg/perside), fluoxetine (5 µg/perside), or saline (0.9%) into NAc daily for 14 days. Each osmotic minipump was attached to a brain infusion cannula. A total volume of 1.0 µL was infused into each side over 15 min, and the osmotic minipumps were maintained in place for an additional 5 min to limit reflux along the injection track. In some experiments, a nonspecific tyrosine kinase inhibitor, K252a (5 µg/perside), was co-infused bilaterally with d-serine, and K252a was infused bilaterally 20 min before d-serine infusion.

### High-performance liquid chromatography analysis (HPLC)

Tissue samples were weighed after extraction, and homocysteine as an internal standard was added. After that, samples were homogenized in 10 volumes of 5% trichloroacetic acid (TCA), and the homogenates were centrifuged at 18,000×*g* at 4 °C for 30 min. To remove TCA, the supernatants were washed three times with a water-saturated diethylether. The resultant samples were used for HPLC analysis. Amino acid enantiomers were separated by HPLC using a carbon 18 reverse-phase column (250 mm) (Knauer, Advanced scientific instruments, Germany) with fluorimetric detection after derivatization with N-isobutyryl-l-cysteine and O-phthalaldehyde (Tomassoni-Ardori et al. 2019). The Nisobutyryl-l-cysteine/OPA derivatives were immediately applied to HPLC system (Knauer, Advanced scientific instruments, Germany). Mobile phase was 8% MeCN in 0.1 M sodium acetate buffer (pH 6.0). Amino acids were separated isocratically for 37 min, and then the column was eluted with 50% H_2_O/50% MeCN for 5 min. After that, the column was rebalanced for 15 min under the initial conditions. The flow rate was 0.125 mL min^−1^. Fluorescence detection of each amino acid derivative was conducted at 443 nm with excitation at 344 nm. The absolute d-serine levels referring to the internal standard were calculated, and data were related to the wet weight of initial tissue samples.

### Chronic social defeat stress

The social defeat stress model of depression was performed according to our previously reported methods (Jiang et al. 2020). Adult male C57BL/6J mice were the subjects, and CD1 retired breeders were the aggressors. Each C57BL/6J mice was exposed to different aggressive CD1 mice each day for 10 min over a total of 14 days. After 10-min contact, the defeated C57BL/6J mice were separated from CD1 aggressors for the next 24 h by plastic dividers containing holes, where they were exposed to chronic defeat stress in the form of threatening. Undefeated control mice were handled daily. Twenty-four hours after the last stress, defeated C57BL/6J mice were subjected to social interaction test and sorted into either susceptible or resilient phenotype based on interaction scores. All CSDS-resilient mice were removed. Then, CSDS-susceptible mice were housed individually and received daily injections of tested drugs or vehicles for another 14 days. After that, various behavioral tests were performed.

### Forced swim test

The FST test was carried out according to previously reported methods with slight modification (Zhong et al. 2020), and separate groups of C57BL/6J mice were used for this test. Briefly, 30 min after a single injection, mice were individually placed in a glass cylinder (height 45 cm, diameter 20 cm) filled with 25 °C water to a depth of 15 cm for 6 min. All mice were forced to swim for 6 min, and immobility duration during the last 4 min interval of the test was recorded by an investigator blind to the study. The water was replaced after each trial. A mouse was judged to be immobile when it remained floating in water without struggling and only making movements necessary to keep the nose above the water. The observers were unaware of the treatment of mice.

### Tail suspension test

The TST is a widely used test for assessing potential antidepressant-like medications. The TST test was measured according to methods described previously (Zhong et al. 2020), and separate groups of C57BL/6J mice were used for this test. Briefly, 30 min after a single injection, the test C57BL/6J mice were suspended 60 cm above the floor for 6 min by adhesive tape placed approximately 1 cm from the tip of the tail. The immobility duration was scored between the 2nd and 6th minute for 4 min by an investigator blind to the treatment. Mice were considered immobile only when they hung passively and were completely motionless, and any mice that did climb their tails were removed from the experimental analysis. The observers were unaware of the treatment of the mice. Means and SEM were calculated for each group.

### Open field test

Spontaneous locomotor activity of experimental C57BL/6J mice was studied in the open field paradigm over a 5-min period (Zhi et al. 2019), and separate groups of animals were used for this test. The open field was divided into two areas, a peripheral area, and a square center. C57BL/6J mice were individually placed in the center of a wooden box (height 40 cm, width 100 cm, length 100 cm). The test room was illuminated with a red bulb (50 W) on the ceiling dimly. For open-field observations, the animals were placed in the central sector 30 min after a single injection. The computer software (EthoVision; Noldus) calculated the velocity of movement, the distance of traveling, and the time spent in the open field center. For each group, the mean value and SEM were calculated. These parameters are thought to reflect locomotor activity and fear or anxiety, respectively. The open field apparatus was thoroughly cleaned between each trial.

### Social interaction experiments

Adult male C57BL/6J mice were the subjects, and CD1 retired breeders were the aggressors. The day after the last injection (day 29), the social interaction test was performed. The social interaction test comprises two trials of 5 min for each. In the first trial (‘target absent’), each mouse was placed into an open-field box and allowed to explore a plastic enclosure placed within the predefined interaction zone. In the second trial (‘target present’), each mouse was returned to the open-field arena containing a plastic enclosure now holding an unfamiliar CD1 mouse. The duration in the interaction zone was obtained using Ethovision XT (Noldus, USA) software (in seconds). In the first trial, the duration in the interaction zone was counted as A. In the second trial, the duration in the interaction zone was counted as B. The ratio of B/A was used as a behavioral index. The smaller its value, the more pronounced the depression symptoms of mice. If the ratio of B/A is less than 1, they are assigned to the depression susceptible group. Otherwise, they are assigned to the resilient group. The open-field apparatus was cleaned after each trial to remove olfactory cues.

### Sucrose preference test (SPT)

This test lasted for 4 days and was carried out according to previously reported methods with some modifications(Li-Wen et al. 2020). On the first 2 days, the C57BL/6J mice were free to drink two bottles containing pure water and 1% sucrose solution, respectively. The position of the two bottles was changed every 6 h to prevent potential location preference of drinking. On the 3rd day, both food and two bottles were deprived for 24 h. On the 4th day, the test lasted for 6 h, with the position of the bottles interchanged and the two bottles weighed before and after the test period. Sucrose preference was calculated as the percentage of sucrose solution ingested relative to the total amount of liquid intake. Drug treatments were not provided during the testing days.

### Western blotting experiments

The experiment was processed according to previous reports with some modifications (Taylor and Posch 2014). Animals were killed the day after behavioral studies. To extract total proteins, bilateral NAc were immediately dissected and homogenized in 10 mM Tris HCl pH 8, 150 mM NaCl, 5 mM EDTA, 1% NP-40, 0.5% sodium deoxycholate, and 0.1% SDS containing Complete Mini protease inhibitor cocktail (Snyder 2000), and then kept on ice for 30 min (Ntoukas et al. 2020). The lysates were centrifuged, and supernatants were collected. Then, the samples were mixed with loading buffer and boiled for 5 min. Protein concentration was estimated using BCA Protein Assay (Pierce). After denaturation, equal amounts of protein samples (30 mg) were separated by 10% SDS/PAGE gel and then transferred to nitrocellulose membranes (Bio-Rad, Hercules, CA, USA). After being blocked with 10% non-fat dried milk powder/Tris-buffered saline Tween-20 (TBST) for 1 h, membranes were incubated overnight at 4 °C with primary antibodies to BDNF (1:500; Abcam, UK), TrkB (1:1000; Abcam, UK), phospho-TrkB-tyr515 (p-TrkB;1:500; Abcam, UK), cAMP response element-binding protein CREB (1:1000; Abcam, UK), phospho-CREB-Ser133 (pCREB; 1:500; Abcam, UK), and β-actin (1:1000; Abcam, UK). Then, primary antibodies were removed by washing membranes three times in TBST. The membranes were further incubated for 2 h at room temperature with IRDye 680-labelled secondary antibodies (1:2000; Santa Cruz). Finally immunoblots were visualized using enhanced chemiluminescence (ECL; Pierce, Rockford, IL, USA). The optical density of bands was quantified using ImageJ software (Packard Instruments BV, Groningen, Netherlands). The results were normalized to the quantity of β-actin in each sample lane. All assays were performed at least three times.

### Electrophysiological recordings

NAc slices were prepared from C57BL/6J mice as previously reports with some modifications (Li et al. 2018). Briefly, brains were rapidly removed, and Sagittal plane brain slices (400 µm thickness) containing NAc were cut using a vibrating blade microtome in ice-cold artificial cerebrospinal fluid (ACSF) containing (mM) 119 NaCl, 3.5 KCl, 1.3 MgSO_4_, 2.5 CaCl_2_, 1 NaH_2_PO_4_, 26.2 NaHCO_3_ and 11 glucose that was bubbled continuously with 95%O_2_–5%CO_2_ to adjust pH to 7.4. After 2 h of recovery at 28 °C, an individual slice was transferred to a submerged recording chamber superfused with oxygenated ACSF at 30 °C at a rate of 3–4 mL·min^**−**1^. Prefrontal cortex-accumbal afferents were stimulated by delivering stimuli through a bipolar stimulating electrode implanted into PFC near PFC-NAc border 0.5–3 mm dorsal to the recording electrode placed in NAc, as we reported(Li et al. 2018). Field excitatory postsynaptic potentials (fEPSPs) were recorded by a microelectrode filled with 3 M NaCl, and the test frequency to evoke fEPSPs was 0.03 Hz. Stimulation intensities were chosen to produce an fEPSP with a slope that was 60% of that obtained with maximal stimulation. Input–output (I/O) relationship for synaptic transmission was recorded by varying the intensity of the single-pulse stimulation. Paired stimuli (25, 50, 75, and 100 ms interval) were delivered, and the paired-pulse ratio (PPR) was calculated as the ratio between the mean slope of the second fEPSPs over the first fEPSPs. The initial slope of fEPSPs was measured and expressed as a percentage change from the baseline fEPSPs level, calculated from the average of the last 20 min of the baseline recording period. LTD was induced by the following protocol: NMDAR-dependent LTD in NAc was induced by a stimulating protocol that consisted of one train of stimulus at 1 Hz (15 min) after 10 min of stable baseline recording. The magnitude of NMDAR-dependent LTD was calculated from fEPSPs recorded after 40 min of LTD induction as the percentage of baseline EPSP slopes (Zhang et al. 2017).

### Statistical analysis

All analyses were implemented using SPSS 24.0 software (SPSS Inc., USA), and data are rendered as mean ± SEM (standard error of the mean). The comparisons between groups were evaluated using one-way ANOVA (post-hoc LSD test) or two-way ANOVA (post-hoc Bonferroni’s test), as appropriate. A value of P < 0.05 was considered statistically significant.

## Results

### The concentration of d-serine in NAc was decreased in depression susceptible mice

**Fig. 1:**
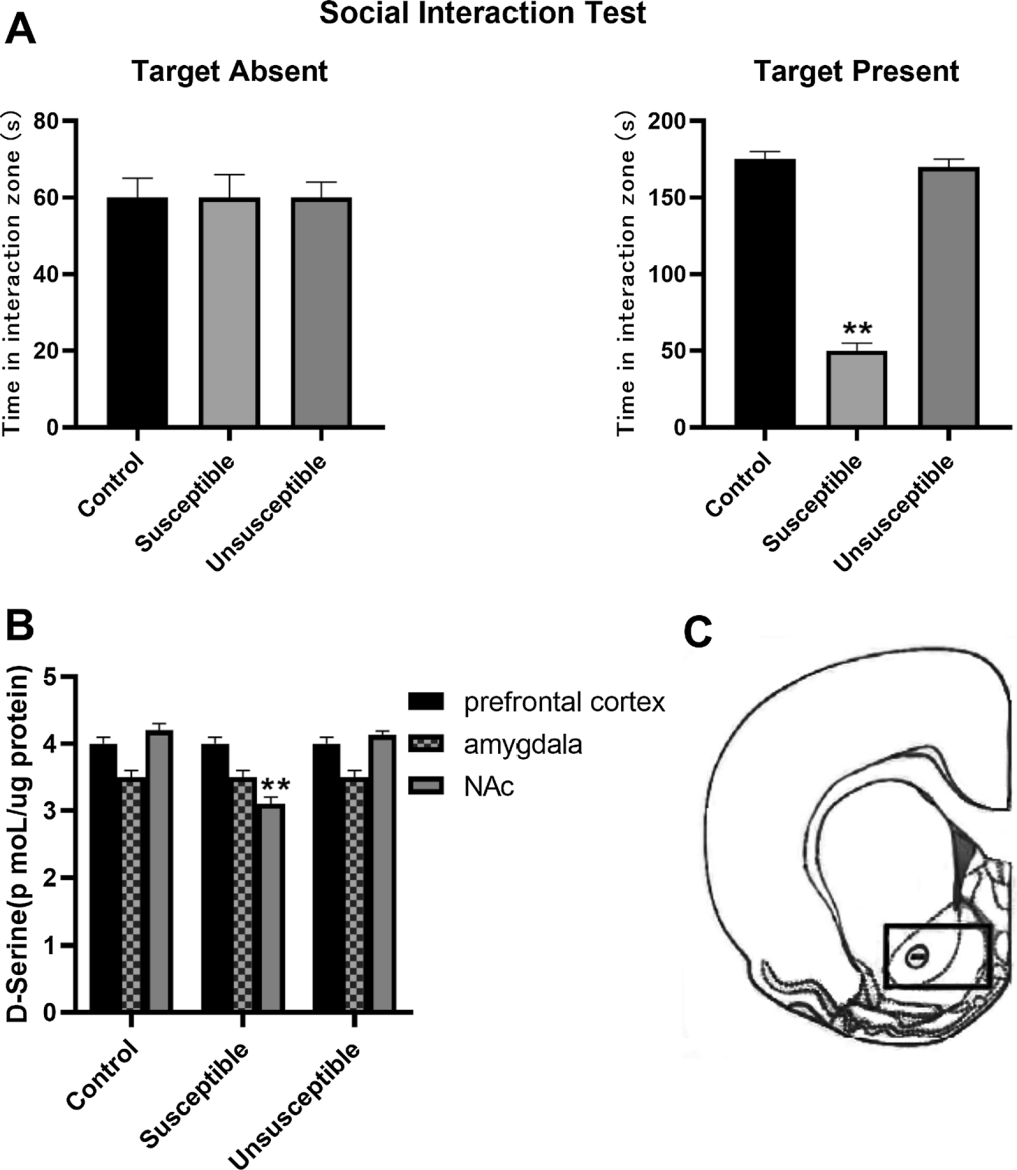
The results of high-performance liquid chromatography demonstrated that d-serine concentration in NAc was decreased in depression susceptible group. **A** Results of behavioral experiments. **B** Average content of d-serine in prefrontal cortex, amygdala, and NAc in normal control group, depression susceptible group, and depression unsusceptible group. **C** Anatomical location of NAc. The data are expressed as mean ± SEM (n = 12); **P < 0.01, significantly different from control. Comparisons were made by one-way ANOVA followed by a post-hoc LSD test

After a brief (10 min) daily exposure to a highly aggressive resident mouse for 14 days, the mice were divided into depression susceptible group and depression unsusceptible group according to the results of behavioral experiments (Fig. 1A). Subsequently, the prefrontal cortex, amygdala, and NAc of normal control group, depression susceptible, and depression unsusceptible groups were extracted. Finally, the average content of d-serine in three different brain regions was calculated using HPLC. The results demonstrated no significant difference in d-serine content in the prefrontal cortex and amygdala of the normal control group, depression susceptible group, and depression unsusceptible group. However, compared with the normal control group and the depression unsusceptible group, d-serine content in NAc of depression susceptible group was significantly reduced (n = 12, P < 0.01 vs. control or unsusceptible; Fig. 1B). Figure 1C is the anatomical location of NAc. The results indicated that d-serine concentration in NAc was decreased in depression susceptible group.

### Antidepressant-like effects of d-serine in mice

**Fig. 2:**
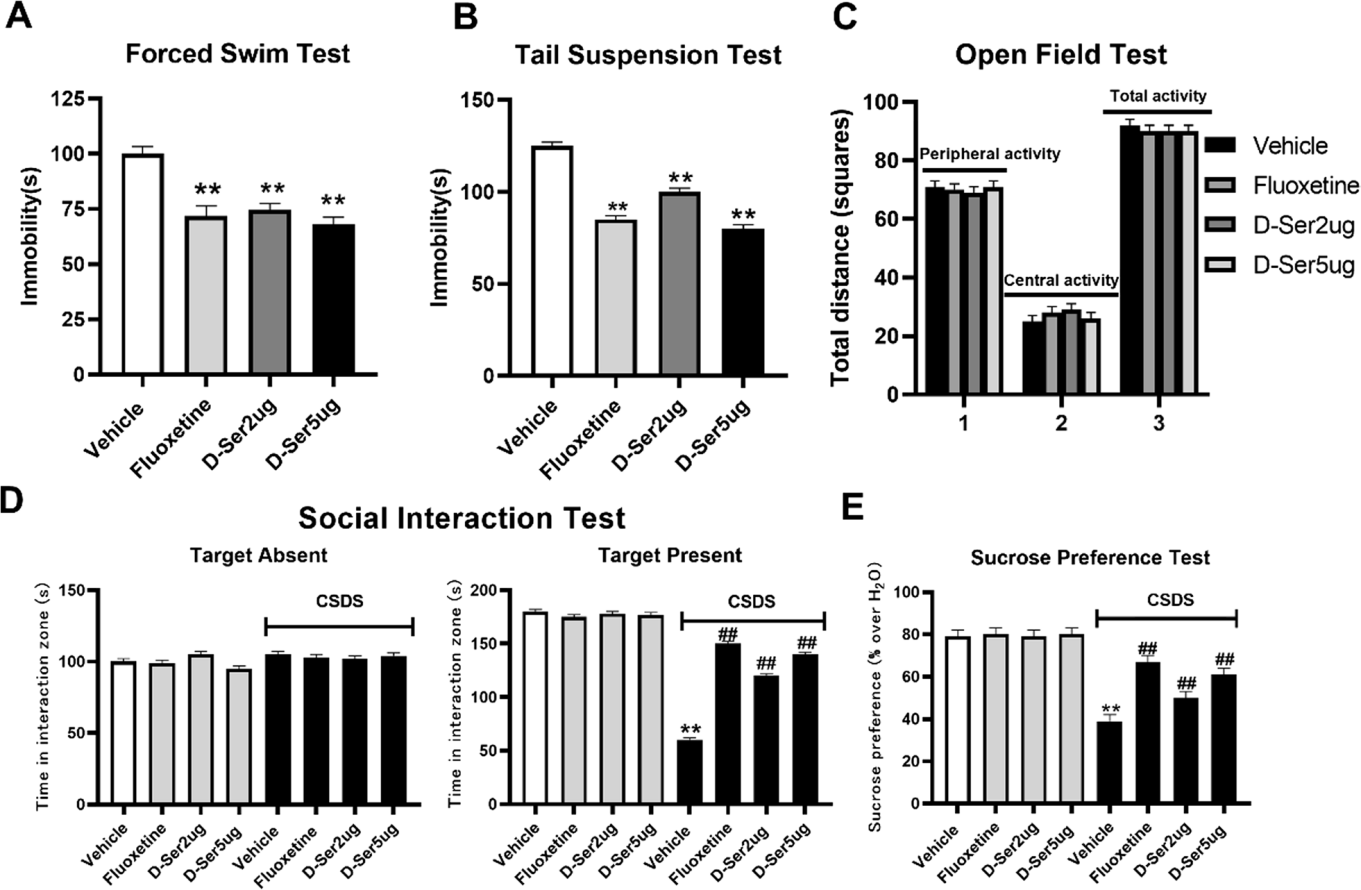
d-Serine exhibits antidepressant-like effects in FST, TST, and CSDS models. C57BL/6J mice were injected into bilateral NAc with a single dose of vehicle (Control), fluoxetine (5 µg/perside), or d-serine (2, 5 µg/perside). The behavioral tests were conducted 30 min after the injection. Meanwhile, CSDS-stressed mice received daily injections (injected into bilateral NAc) of vehicle (Control), fluoxetine (5 µg/perside), or d-serine (2, 5 µg/perside) for 14 days, behavioral tests were then conducted. The vehicle referred to 0.9% saline (injected into bilateral NAc). **A** d-Serine observably reduced the immobility time of C57BL/6 J mice in FST. **B** d-Serine observably reduced the immobility time of C57BL/6J mice in TST. **C** d-Serine administration had no effects on spontaneous locomotor activity of mice in the open-field test. In C, "1" represents peripheral activity, "2" represents central activity, and "3" represents total activity. **D** The antidepressant-like outcomes of d-serine in the social interaction test. CSDS + d-serine mice spent significantly more time concerned with social interaction than CSDS + vehicle mice. **E** The antidepressant-like outcomes of d-serine in the sucrose preference test. CSDS + d-serine mice showed significantly higher sucrose preference than CSDS + vehicle mice. Fluoxetine was used as a positive control. The data are expressed as mean ± SEM (n = 12); *P < 0.05, **P < 0.01, significantly different from control + vehicle; ^##^P < 0.01 vs CSDS + vehicle. Comparisons were made by one-way ANOVA followed by a post-hoc LSD test

FST and TST in mice are widely used for detecting potential antidepressant-like activity (Wang et al. 2020). d-Serine was injected into bilateral NAc, with fluoxetine used as a positive control. Analysis indicated that compared with vehicle group, d-serine (2, 5 µg/perside) or fluoxetine (5 µg/perside) treatment significantly decreased the immobility time in FST (Fig. 2A). Similar to consequences of FST, d-serine administration robustly reduced immobility duration of mice in TST, compared with vehicle group (Fig. 2B). The magnitude of d-serine (5 µg/perside) induced anti-immobility effect was comparable with that of fluoxetine (5 µg/perside).

To exclude the probability that reduced immobility in these tests might result from an increase in spontaneous locomotor activity (Eissa et al. 2020), mice were exposed to the open-field test. We found no essential differences in the number of squares an animal crossed between the center region or the periphery region in all groups (Fig. 2C) and no effects for drug administration. These data indicated that the decrease of immobility observed in the two tests after d-serine disposal was not because of locomotor hyperactivity.

We further utilized CSDS model to characterize antidepressant effects of d-serine, which mimics many depression symptoms in human (Rouhiainen et al. 2019). As displayed in Fig. 2D, all mice spent similar time in the interaction zone during the target-absent trial. CSDS-defeated mice spent significantly less time in the interaction zone compared to vehicle-treated mice when CD1 aggressor was present. Interestingly, 14 days of administration with d-serine significantly reversed CSDS-induced social avoidance behavior, peculiarly at 5 µg/perside, similar to fluoxetine. In addition, CSDS induced a significant decrease in sucrose preference compared with the control group. 14-day administration with d-serine in CSDS-susceptible mice prompted a noticeable increase in sucrose intake (Fig. 2E). Collectively, these results revealed that d-serine produces antidepressant effects.

### Chronic d-serine treatment reversed CSDS-induced increase BDNF signaling pathway in NAc

**Fig. 3:**
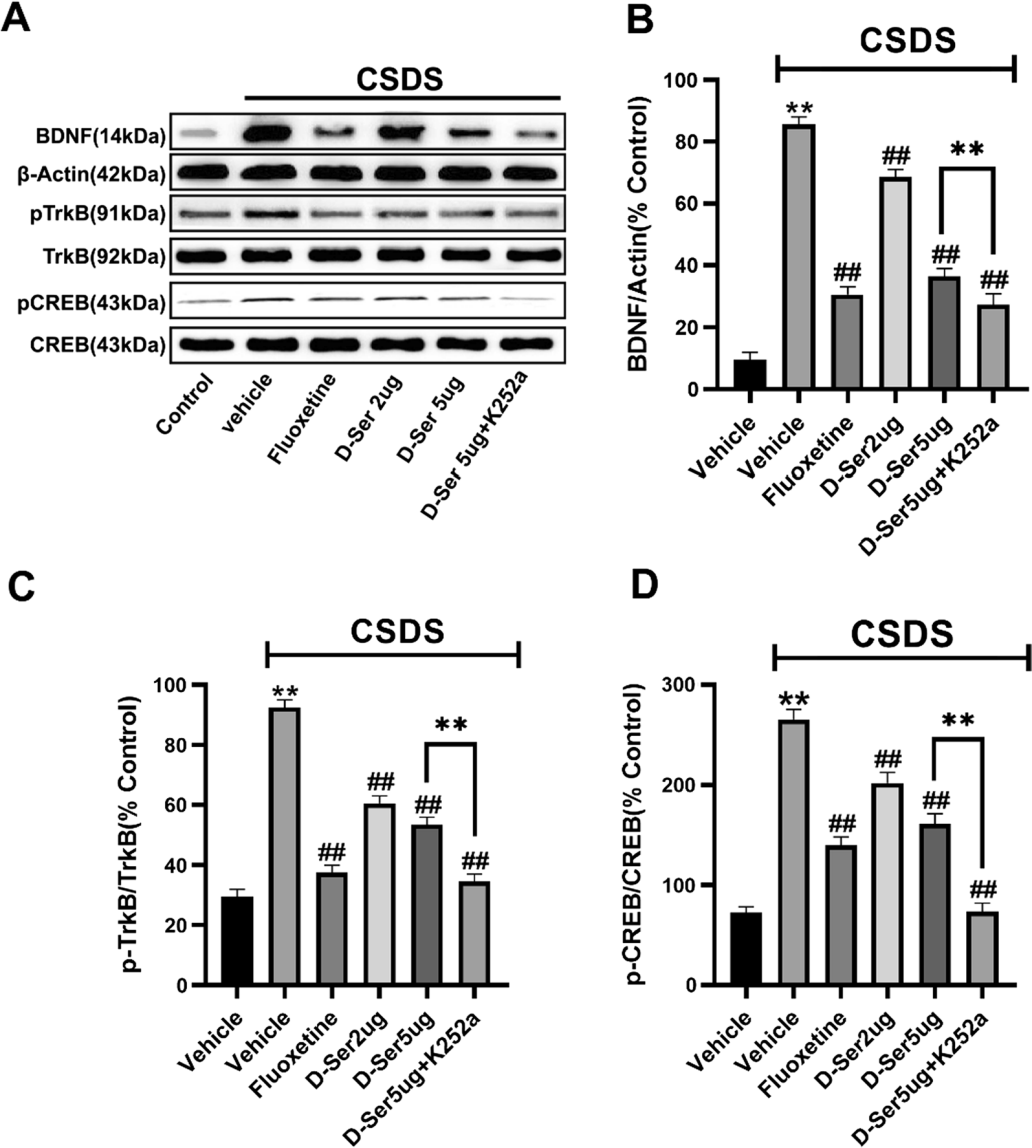
d-Serine administration reverses the CSDS-induced increase in NAc BDNF signaling pathway. Moreover, K252a improves the effects of d-serine on the NAc BDNF signaling pathway. **A** Typical images of our western blotting results. **B**–**D** Our western blotting data displayed that d-serine administration reversed CSDS-induced increase of NAc BDNF, pTrkB, and pCREB protein levels. CSDS + d-serine mice showed meaningfully lower expression of BDNF, pTrkB, and pCREB in NAc than CSDS + vehicle mice. Furthermore, K252a improved d-serine-induced weakness of NAc BDNF, pTrkB, and pCREB expression, as CSDS + d-serine + K252a mice displayed significantly lower BDNF, pTrkB, and pCREB levels in NAc than CSDS + d-serine mice. Data are expressed as means ± SEM (n = 5);**P < 0.01, significantly different from control; ^##^P < 0.01, significantly different from CSDS + vehicle group. Comparisons were made by two-way ANOVA followed by post-hoc Bonferroni's test

To investigate the potential mechanisms underlying d-serine-induced antidepressant effects, we inspected the expression of BDNF signaling pathway protein levels. Data are summarized in Fig. 3A. The BDNF expression level was expressed as a ratio of β-Actin expression. As displayed in Fig. 3, CSDS increased NAc BDNF and pTrkB protein levels compared with the control, similar to reported (Dong et al. 2017). Compared with CSDS group, d-serine treatment significantly decreased their expression. The CREB in NAc is a significant mediator of neural plasticity and the transcription factor for BDNF. d-serine decreased pCREB level in NAc of CSDS mice. Therefore, d-serine treatment prevented stress from higher BDNF, pTrkB, and pCREB in NAc. In contrast, total TrkB and CREB levels were unchanged among all groups. Moreover, d-serine-induced decrease in NAc BDNF expression of defeated mice was also improved by K252a treatment (n = 5; Fig. 3). In line with this, K252a also enhanced d-serine effects on the expression of NAc pTrkB and pCREB (n = 5; Fig. 3). However, the level of total TrkB and CREB protein was not altered. As a result, d-serine-induced changes in BDNF signaling pathway in NAc are involved in mediating depression.

### d-Serine has antidepressant-like effects through restraining BDNF signaling pathway in NAc

**Fig. 4:**
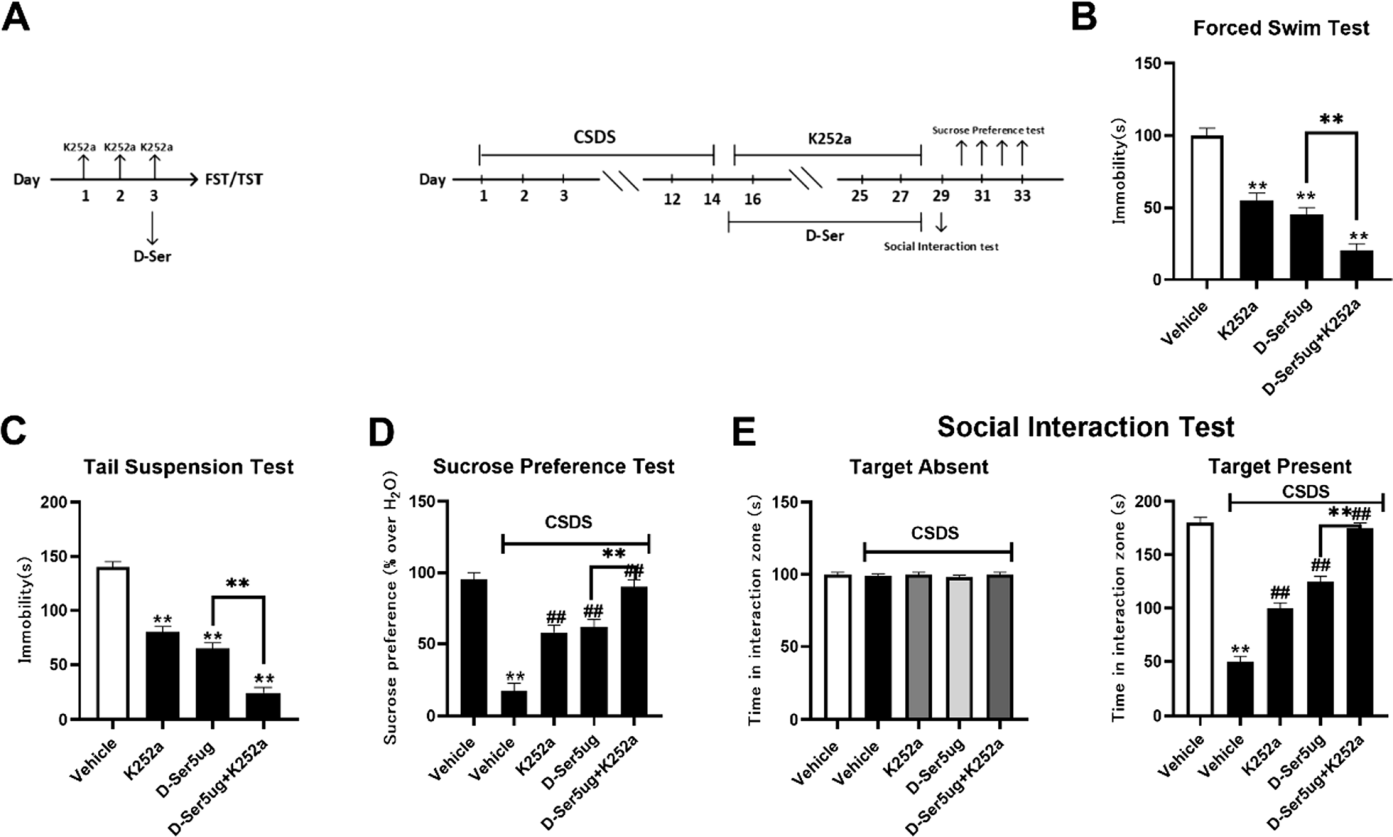
Retardant of BDNF signaling cascade by K252a infusion enhances the antidepressant effects of d-serine. The vehicle refers to 0.9% saline (injected into bilateral NAc). **A** Schematic timeline of experimental steps. After 14 days of CSDS, CSDS-susceptible mice received a daily injection of d-serine (2, 5 µg/perside) and K252a for another 14 days, after which behavioral tests were performed. **B** Pre-infusion of K252a significantly enhanced d-serine-induced reduction in immobility in FST. **C** Pre-infusion of K252a also enhanced d-serine-induced reduction in immobility in TST. **D** CSDS + d-serine + K252a mice showed significantly higher sucrose preference than CSDS + d-serine mice. **E** Co-treatment of d-serine with K252a also enhanced antidepressant effects of d-serine in the social interaction test. CSDS + d-serine + K252a mice showed significantly higher social interaction than CSDS + d-serine mice. The results are expressed as means ± SEM (n = 12), **P < 0.01 vs control; ^##^P < 0.01 vs CSDS + vehicle. Comparisons were made by two-way ANOVA followed by post hoc Bonferroni's test

To determine the relationship between the antidepressant effect of d-serine and BDNF system, K252a, a potent pharmacological inhibitor of BDNF receptor TrkB, was applied. Figure 4A is the schematic timeline of the experimental steps. C57BL/6J mice were daily infused with K252a (5 µg/perside, injected into bilateral NAc) for three days, then treated with d-serine (5 µg/perside), and followed by FST or TST. It was found that infusion of K252a alone decreased the immobility of test mice in FST and TST. K252a pretreatment significantly facilitated the antidepressant effects of d-serine in FST and TST (Fig. 4B, C). Furthermore, CSDS-stressed mice were co-treated with d-serine (5 µg/perside) and K252a (5 µg/perside) for 14 days, and behavioral tests were then implemented. Co-treatment K252a with d-serine significantly enhanced the antidepressant-like effects of d-serine in sucrose preference test (Fig. 4D) and social interaction test (Fig. 4E). These results indicate that K252a enhances the antidepressant effects of d-serine.

## The role of d-serine in synaptic plasticity in NAc of depressed mice

### Impaired synaptic plasticity in NAc of depressed mice

Growing evidence supports the notion that synaptic adjustability in NAc and its connected circuitry plays a critical function in various forms of reward-dependent learning (Grueter et al. 2013). Furthermore, current studies have certified that synaptic molecular adaptability exists in the neurons of NAc that underlie susceptible and plasticity responses to chronic stress. Three forms of synaptic plasticity at prefrontal cortex-accumbal glutamatergic synapses—paired-pulse facilitation (PPF), input–output relationship (I–O), and LTD—were inspected in sagittal slices by us to identify the adjustment of synaptic plasticity by d-serine.

**Fig. 5:**
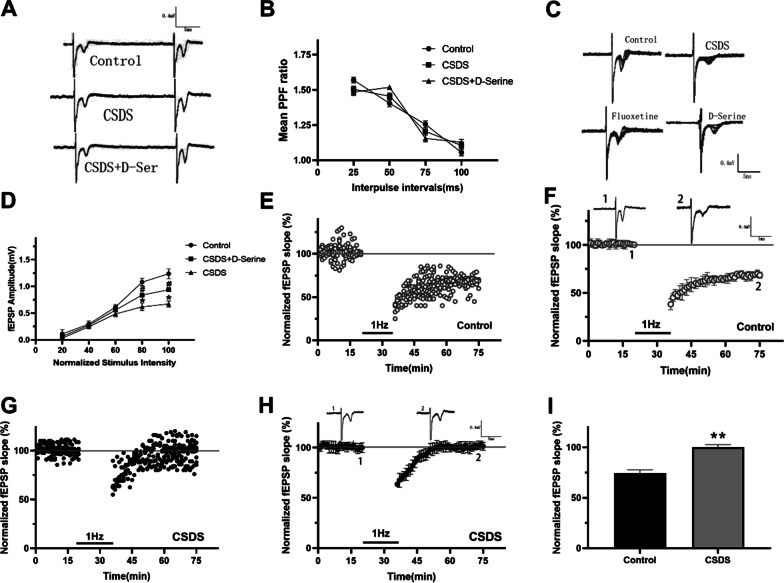
NMDAR-LTD of prefrontal cortex-accumbal glutamatergic synapse was disrupted in CSDS mice. **A** Prefrontal cortex-accumbal field potential recordings of acute NAc slices reveal representative field excitatory postsynaptic potentials (fEPSPs) demonstrated respective recordings from sample experiments at 50 ms inter-pulse interval. **B** Paired-pulse facilitation (PPF) was recorded by changing intervals between pairs of salts. Paired-pulse ratios, that is to say, slope of fEPSPs no. 2/slope fEPSPs no. 1 were alike among control, CSDS, and d-serine treated slices (n = 8). **C** Prefrontal cortex-accumbal field potential recordings of acute brain slices exhibit the characteristic superposed fEPSPs by adding stimulation intensity. **D** Input–output curves illustrating the correlation between magnitudes of stimulation and evoked reaction for fEPSPs recorded from slices of control and CSDS mice. The CSDS decreased fEPSPs in NAc significantly, compared with control mice (n = 8). The d-serine treated slices increased fEPSPs in NAc significantly, compared with CSDS mice (n = 8). **E** Individual experiment traces showed that LTD was induced in control slices by low-frequency stimulation (LFS). The LFS consists of a 1-Hz, 900-pulse train, which has been used to cause NMDAR-LTD in prefrontal cortex-NAc synapse. **F** Averaged data demonstrated that LTD was induced in control slices by LFS. **G** Individual experiment traces showed that LTD was induced in CSDS slices by LFS. **H** Averaged data showed that LTD was induced in CSDS slices by LFS. **I** The column diagram demonstrated the level of LTD 40 min after LFS from control mice and CSDS mice. The NMDAR-LTD was impaired in CSDS mice compared with control mice. **P < 0 .01 vs. control group. The superimposed fEPSP in the upper portion is recording from representative experiments taken at the time indicated by the number. Each point was normalized mean ± SEM of eight slices

The PPF is a subtle estimate of the probability of transmitter liberation. Our results revealed that CSDS produced no outcomes on PPF, hinting at the lack of noticeable change in presynaptic action (Fig. 5A, B). To determine whether synaptic effectiveness was varied with chronic stress exposure and antidepressant administration, input–output (I–O) relationships for field excitatory postsynaptic potentials amplitude were compared. Electrophysiological recordings showed a critical reduction in the amplitude of field excitatory postsynaptic potentials in CSDS group as compared with control group (Fig. 5C, D). Moreover, chronic d-serine administration reversed the damage of basic prefrontal cortex-accumbal glutamatergic synapse transmission from CSDS mice (Fig. 5C, D). d-Serine is suggested to improve the basic synaptic damage in CSDS mice. As shown in Fig. 5E–I, NMDAR-dependent LTD of prefrontal cortex-accumbal glutamatergic synapse was impaired in CSDS mice, although it was normal in control mice. It is indicated that depressed mice’s synaptic plasticity in NAc was impaired.

### d-Serine modulates depression-mediated changes in synaptic plasticity in NAc

**Fig. 6:**
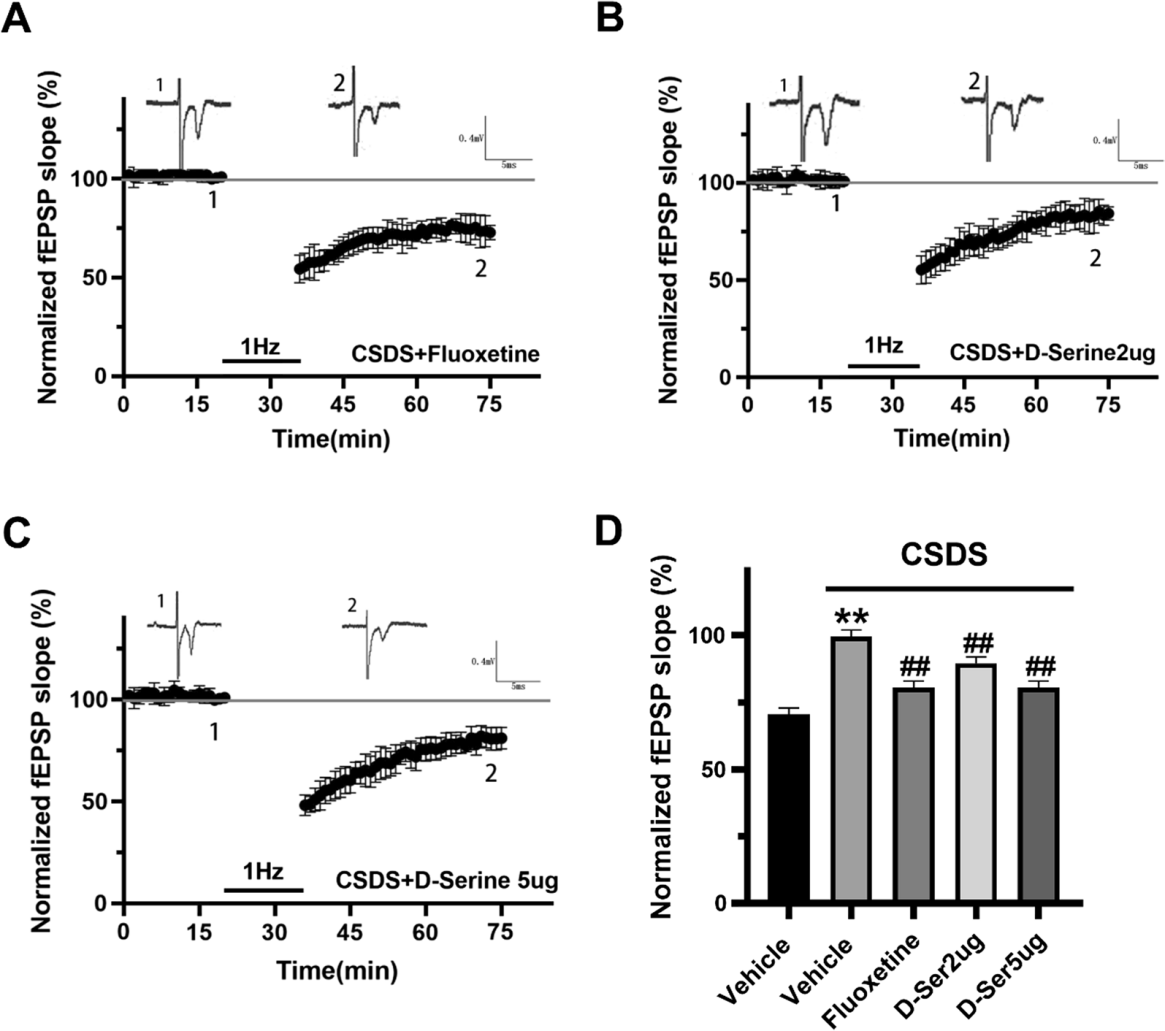
d-Serine administration reversed the impaired NMDAR-dependent-LTD in NAc of CSDS mice. **A**–**C** Averaged data demonstrated that LTD was induced in CSDS + fluoxetine mice, CSDS + d-serine (2 µg/perside) mice, and CSDS + d-serine (5 µg/perside) mice by LFS. **D** The column diagram demonstrated the level of LTD 40 min after LFS from control mice, CSDS mice, CSDS + fluoxetine mice, CSDS + d-serine (2 µg/perside) mice, and CSDS + d-serine (5 µg/perside) mice. Fluoxetine or d-serine administration reversed the damaged NMDA-LTD in NAc of CSDS mice. **P < 0.01 vs. control group; ^**##**^P < 0.01 vs. CSDS group. The superimposed fEPSP in the upper portion is recording from representative experiments taken at the time indicated by the number. Each point was normalized mean ± SEM of eight slices

Furthermore, NMDAR-LTD in NAc were compared in control, CSDS, CSDS + fluoxetine and CSDS + d-serine (2, 5 µg/perside) groups. Chronic fluoxetine or d-serine treatment significantly increased the amplitude of LTD and reversed the impaired NMDAR-LTD in NAc of CSDS mice (Fig. 6A–D). Overall, the electrophysiological data indicated that synaptic plasticity in NAc is disrupted in CSDS mice, reversed by d-serine treatment, backing a role for NMDAR-LTD in NAc in depression.

## Discussion

The following are the most significant findings from this study. d-Serine initially exhibited antidepressant-like properties in FST, TST, and CSDS models. Following that, chronic d-serine treatment could restore a stress-induced increase in NAc BDNF signaling cascade. Thirdly, decreased d-serine levels in NAc result in impaired synaptic plasticity. Together, we have demonstrated for the first time that d-serine protects against depression by inhibiting BDNF signaling pathway and regulating synaptic adaptations in NAc. This discovery is fascinating and intriguing since it identifies a novel antidepressant.

d-Serine can pass through the blood–brain barrier, and we have simultaneously completed the in vivo experiment of intraperitoneal injection. Our data demonstrated that d-Serine possesses characteristics similar to those of conventional antidepressants in FST and TST. In these tests, mice were placed under stressful circumstances from which they could not escape and eventually became powerless and immobile, a state similar to human depression that could be reversed by antidepressant pharmaceuticals. As expected, the decrease in immobility in FST and TST induced by acute d-serine injection in mice was not accompanied by an increase in locomotor activity, implying that d-serine could have therapeutic potential against depression. We further utilized CSDS model to confirm d-serine outcomes, as CSDS model exhibits great predictive validity for symptomatology of stress-related disorders like depression (Zhang 2020). The sucrose preference test is an indicatrix of anhedonia-like behavioral alterations. Our findings demonstrated that d-serine administration fully renovated the sucrose preference of CSDS-stressed mice to normal levels. Another prominent symptom of depression is social delusion, meaning that depressed rodents are passive and tempting to flee from the world. Our results indicate that d-serine administration also reversed the social avoidance of CSDS-stressed mice, similar to fluoxetine. Collectively, these discoveries demonstrate that d-serine could be developed into a novel antidepressant.

Moreover, the discovery that d-serine could reverse CSDS-induced effects on NAc BDNF signaling cascade is novel. This research is the first to provide experimental evidence representing that d-serine affects BDNF system in NAc. This research found that chronic d-serine administration down-regulated level of BDNF phosphorylated TrkB and phosphorylated CREB in NAc of stressed mice to the basal level of saline-treated mice. Moreover, antidepressant effects induced by d-serine administration were also facilitated by co-treatment with K252a, a potent inhibitor of BDNF receptor TrkB.

Synaptic plasticity is regarded as a cellular substrate of learning and memory (Kamalova et al. 2020). d-Serine plays a significant role in activating NMDAR-dependent synaptic plasticity (Cha et al. 2020). NAC neurons are among those that lightly express LTD, which is susceptible to stress. We found that d-serine facilitated LTD. d-serine in NAc modulates drug addiction through synaptic plasticity (Liu et al. 2016). It is well known that drug addiction and depression are regulated by NAc, which are diametrically opposed behaviors. Our results support that LTD was markedly damaged in NAc of CSDS mice and was reversed by a chronic d-serine injection. LTD is induced by low rises of intracellular Ca^2+^ concentration (Bezprozvanny and Hiesinger 2013). Reduced Ca^2+^ influx at postsynaptic level with hypofunction of NMDAR could persuasively explain the damage of LTD in our CSDS model. So, our results manifested that decreased d-serine levels in NAc of CSDS mice hindered the induction of NMDAR-dependent LTD. By electrophysiological techniques, our consequences sustain NMDAR-LTD molecular substrate that convey this lack of plasticity in CSDS and could result in new targets for antidepressants.

In summary, this study's findings indicate that d-serine possesses antidepressant-like effects in mice, appearing to be mediated through down-regulation of NAc BDNF-TrkB signaling pathway. In addition, it indicates that chronic social defeat stress treatment can alter the metabolism of d-serine in the NAc, leading to reduced d-serine levels and impairment of NMDAR dependent LTD. Moreover, increasing d-serine content in NAC can improve depression. Therefore, this provides new insight on pharmacological outcomes of d-serine and illuminating the development of new antidepressants with higher effectiveness and fewer side reactions. Furthermore, our related experiments, such as SR-shRNA, DAAO-shRNA, NMDAR blocker MK-801, NR2B-shRNA, and so on, are in progress concurrently.

## Conclusions

Our results provide the first evidence that d-serine exerts antidepressant-like effects in mice mediated through restraining BDNF signaling pathway and regulating synaptic plasticity in NAc, implying that d-serine may be an effective therapeutic agent toward depression.

## Data Availability

All data and materials are available.

## Abbreviations

NMDARs: N-methyl-d-aspartate receptors
NAc: Nucleus accumbens
CSDS: Chronic social defeat stress
HPLC: High performance liquid chromatography
BDNF: Brain-derived neurotrophic factor
TrkB: Tyrosine receptor kinase B
CREB: CAMP response element-binding protein
FST: Forced swimming test
TST: Tail suspension test
LTD: Long-term depression
fEPSPs: Field excitatory postsynaptic potential
LFS: Low-frequency stimulation
